# Examining the effects of parental migration on youth mental health and substance use: a qualitative study in rural Yucatán, México

**DOI:** 10.3389/fpsyt.2024.1368619

**Published:** 2024-05-14

**Authors:** María Luisa Zúñiga, Kayla Mulholland, Pedro Lewin-Fischer, Isela Martinez San Román, Lidiane Toledo, Lianne Urada

**Affiliations:** ^1^ School of Social Work, College of Health and Human Services, San Diego State University, San Diego, CA, United States; ^2^ Sección de Lingüística, Centro INAH Yucatán, Instituto Nacional de Antropología e Historia, Mexico City, Mexico; ^3^ Oswaldo Cruz Foundation (Fiocruz), Rio de Janeiro, Brazil

**Keywords:** parental migration, youth left behind, adolescent health, youth alcohol consumption, substance use, remittances, youth resilience, Mexico

## Abstract

**Background:**

Parental migration is common in Mexico and Latin America, where individuals pursue work to improve their family’s economic opportunities and children remain home in their community under the care of the remaining parent or extended family. A research gap remains about the impact of parental migration on mental health and substance use in children who remain at home. The current study explored risk and resilience factors relating to mental health and substance use among Mexican youth remaining at home when one or more parents migrate.

**Methods:**

This qualitative study applied attachment theory and thematic analysis to analyze 26 in-depth interviews with youth (17-21 years old), parents, and a focus group with high-school teachers in a town with history of migration both domestically and internationally (Yucatan, México).

**Results:**

Respondents across groups perceived that parental migration was related to 1) less parental/caregiver oversight and support due to family demands on the remaining parent and 2) the deterioration of youth mental health. Lack of youth oversight and the poor mental health of youth were perceived as drivers of youth seeking out and consuming alcohol and substances. In terms of parental remittances, youth reported observing among their peers increased access to material goods such as clothing and technology (e.g., smartphones) and increased access to alcohol. Resilience factors included parental awareness of the role of good communication with youth and teachers and youth access to and utilization of self-care resources such as mutual aid meetings for substance use recovery.

**Conclusion:**

Poor mental health and substance use among youth and parents were perceived to be related to parental absence, stressors on the remaining parent or family, and undermined healthy parent-child attachment. Youth themselves are a source of insight for recommendations on interventions to reduce youth isolation and substance use risk. We recommend the intentional engagement of youth in developing intervention research and tailoring evidence-based interventions to mitigate parental absence’s impact and promote parent-child attachment for youth and families remaining at home.

## 1 Introduction

Parental absence resulting from economic migration is a complex, global phenomenon with profound implications for the well-being of children and families who remain in the community of origin (1–3). Among the world’s 184 million people who have migrated from their homes to another country, about 80% (147 million) are economic migrants looking for work to improve the quality of life for themselves and their families (4). In their comparative research on parental migration and parental absence in Latin America, DeWaard and colleagues (2018) noted that among international migrants of Mexican origin (N=3,743), about 34% had a child under the age of 18 in the home at the time of their departure; this was slightly lower than the 36% with a child in the home among those with domestic migration experience (N=2,324) (5). Mexico has a long history of economic migration to the U.S., its neighbor to the North, and is the second largest emigrant population in the world after India: approximately 11 million people of Mexican origin live in the U.S (6). Indeed, globally, Mexico is the country with the second-highest receipt of remittances, following India (6): According to the Banco de México, in 2023, Mexico received USD $63.3 billion in remittances (7), with almost all (95%) of remittances to Mexico coming from the U.S (8).

The effects of parental absence on child well-being and mental health, including the positive and negative impacts of remittances, are also evident, and research on parental migration underscores the complexity of factors that affect child health outcomes (3, 9, 10). Benefits of parental migration and remittances to families remaining in the community of origin have been documented and include improved economic wellbeing of the family, access to education, and transmission of new perspectives and gender equity (1, 2). Parental migration has also been associated with child misbehavior, school drop-out, and poor mental health outcomes in youth who remain in communities of origin, including the onset and aggravation of depression, suicidal behavior, and substance use disorders (9, 11–13). Feelings of social isolation, loneliness, and abandonment are also common among those youth (3, 14). Recent work by Carranza (12) and Ciborowski and colleagues (13) sheds much-needed light on the circumstances faced by children and families in Latin America, Nicaragua, El Salvador, and Guatemala, respectively, who remain in the community of origin when a parent or partner migrates. Globally, among systematic reviews and meta-analyses on the effects of parental migration on mental health and substance use among children who remain, few studies emanate from Latin America (9, 10). Additional research is needed to provide a more nuanced understanding of youth substance use risk and factors that may promote access to and consumption of alcohol and other substances in the Latin American context of parental migration. Alcohol and substance use among youth places them at higher risk for problematic use (e.g., substance use disorder) as they age into adulthood (15).

In Mexico, alcohol use is highly prevalent and deeply embedded in its history and cultural, social and family norms (16), where about 60% of the adult population (ages 18-65) has a family history (e.g., one or both parents who drank) of alcohol use (17). In Western Mexico, it has been found that over 61% of 12-17-year-olds have already begun drinking alcohol (18). While alcohol is considered to be an important element of social gatherings and family celebrations, there is increasing interest by parents for interventions that reduce underage drinking (19).

We undertook the current qualitative study to explore in-depth risk and protective factors that may influence mental health, substance use and other risk behaviors among youth who remained in their community of origin. Onward, this paper will focus on youth with an immigrant parent, where we define “youth” as individuals who are 18 to 21 years of age and considered older adolescents by the American Academy of Pediatrics (20). We engaged with community members, administrators and staff of the high school in the rural Maya indigenous community of Tunkás in Yucatán, México, where many students experience the migration of one or both parents. Examining the factors that contribute to poor mental health and substance use disorders, as well as factors that promote resilience among youth who remain in their community, can inform future research and guide the development of interventions aimed at promoting the well-being of young people with migrating parents.

## 2 Methods

This qualitative study was conducted using the principles of Community-engaged Research and Citizen Science (21) with community members in the municipality of Tunkás, Yucatán, México, a largely rural agricultural community of indigenous Maya. Citizen science is an approach to informing research through the leadership and active participation of community members in community-led projects and in the research process through partnerships with researchers (21). Based on earlier community feedback obtained when the investigators (Lewin Fischer and Zúñiga) met with community members to discuss the impact of migration on the community, teachers in the group noted concerns about youth risk behavior in the context of parental migration. Informed by this community feedback, the research team (Fischer, Martínez SanRomán & Zúñiga) later planned the current study in collaboration with the faculty and administration of Tunkas’s only high school, an agricultural high school named *Centro de Bachillerato Tecnológico Agropecuario* (CBTA) No. 284, “Gral. Salvador Alvarado Rubio”, Tunkás, Yucatán (México). This study was approved by the Human Research Protections Program at San Diego State University. The authors wish to note that although the use of the term “left-behind children’’ is common in the parental migration literature, we feel that the term may imply intentional parental desertion of the child, a fact that is not known for our study population. When possible, we therefore describe youth as remaining ‘at home’ or ‘in the community of origin’ versus ‘left behind’ in order to not stigmatize parents who migrate.

Members of the research team have been working with the community of Tunkás since 2006, where household surveys had been conducted every three years through a collaboration between researchers and students in San Diego, California (U.S.) and Mérida, Yucatan (Mexico) (described in Cornelius, W. A.(Eds.), 22). Tunkás was selected as the study site because it is a community that is impacted by domestic and international migration where it is common for members to have experience with both types of migration. In addition, ongoing researcher relationships with members of the community and feasibility of conducting household surveys with the community of Tunkás were factors that guided initial work in Tunkás. The current study is a continuation of this work and was based on community feedback and recommendations for future research on youth risk and migration that members of the study team obtained during presentations of research findings to members of the community as part of ongoing Community Based Participatory Research (February 2013).

### 2.1 Setting

Population estimates from 2020 indicate that Tunkás has 3,684 inhabitants, with 35% under the age of 19 and 20% over the age of 55 (23). Tunkás is a high migrant-sending community with economic migration [e.g., domestic (national) migration to tourist areas (e.g., Mérida and Cancún) and international migration to the U.S.]. Most families have had at least one member migrate to the U.S. or to another region of Mexico (22). When a parent migrates, children often stay with the other parent, older sibling, or extended relatives, including aunts, uncles, and grandparents. Migration generates important resources for families. In 2023, the municipality of Tunkás received remittances of about USD $286,000 (7).

### 2.2 Recruitment

Our inclusion criteria for parents of youth encompassed individuals with direct migration experience or those with a spouse who had migrated, either domestically or transnationally. Our inclusion criteria for youth included being between 17-21 years old and having at least one parent migrate at any time (domestically or transnationally). Youth who had a parent who migrated but returned were also eligible.

### 2.3 Recruitment procedures

As part of the Community Based Participatory Research nature of this study, we first established a strong working relationship with the CBTA school administration and teachers, who guided us on the best way to reach students and parents. They also provided feedback on the interview guide questions.

1. CBTA high school teachers generated an internal list of students with at least one parent who had migrated (this list was not revealed to the investigators). 2. Teachers then reached out to and informed potential youth and parent participants about the study and briefly explained the study to them. 3. Teachers asked students and parents for their verbal permission to provide their contact information to our field research team (MLZ, PLF, IM). 4. Members of the field research team (MLZ, PLF, IM) received a list of students and parents who agreed to be contacted about the study. 5. They then contacted each student and parent, explained the study and administered the informed consent procedures, including parental assent for one person. 6. Researchers then made an appointment with each consented person to interview at a date and time convenient for the participant.

A majority of youth participants (11 of 12) were 18 years of age or older at the time of the interview; many of these participants did not live with their parents. (see **Table 1** demographics). In order to obtain additional parent perspectives, we adapted our recruitment outreach to include a request for referrals from parents who were interviewed to recommend other community parents with family migration history who may be interested in the study. Parental participant referrals occurred in at least two instances and all persons who were contacted consented to participate.

**Table 1:** Youth Demographic Information.

Unique ID	Gender	Age at time of survey (2015)	With whom youth currently lives	Where youth's father currently lives
1	Male	18	Both Parents	Tunkás
2	Female	19	Grandmother	Cancún
3	Female	18	Sister	Mexico City
4	Male	21	Godfather	San Antonio Chuc (In municipality of Tunkás)
5	Female	(missing)	Mother’s partner	Cancún
6	Male	18	Grandmother	Mérida
7	Female	21	Both Parents	Izamal
8	Male	18	Mother	Playa del Carmen
9	Female	19	Brothers/ Sisters	Cenotillo
10	Female	18	Grandparents	Quintana Roo
11	Female	18	Mother	Cancún
12	Female	17	Mother	Inglewood, California

### 2.4 Response rate

Initial contact by teachers: Because the list of potential participants who were contacted by the teachers was unknown to the field research team, we do know the number of persons who preferred not to be contacted. Response rate for study consent was 100%: Of the contacts made by the field research team, all who had agreed to be contacted also consented for the interview.

We conducted 23 in-depth interviews with the following key participants: (i) 11 parents (10 females, one male) who had children attending the high school; and (ii) 12 students aged 17-21 (8 females and 4 males). Participants did not see the interview guide questions ahead of time. Although we interviewed parents and children from the same household, we also interviewed students whose parents were not interviewed. In order to gain additional insights into student experiences with parental migration, we conducted one focus group with teachers and staff (n = 7) from the CBTA high school. All participants provided voluntary informed consent, and all youth under the age of 18 provided additional parental consent to participate in the study.

Data were collected via face-to-face interviews by trained bilingual (Spanish-English) and bicultural researchers between June 17 and July 1, 2015, using a semi-structured interview guide; sample questions from the youth interview guide can be found in **Table 2**. Interviews were conducted in Spanish in a private location that was convenient for the participant (e.g., unoccupied school classrooms and homes where the interview could be conducted privately). Each interview lasted approximately 40–60 minutes and was tape-recorded, transcribed verbatim, and analyzed in Spanish in order to maintain the linguistic nuances within the interviews. The translated quotes in this paper underwent additional review for translation accuracy by a bilingual research team member. The quotes encompass participants’ perceptions of migration-related themes observed in their own experiences and those of others (e.g., peers, parents, students).

**Table 2:** Sample youth in-depth interview questions.

What youth experience when a parent migrates?
Migration to the United States or other places like Quintana Roo can affect members of the family in different ways, such as changes in family responsibilities, daily routines, among other things. Q1. In general, based on what you have observed, in what ways is the family impacted when a father migrates? What about when a mother, or both parents, migrate? Q2. Specifically thinking about the experiences of young people, such as yourselves, in which ways do you think that migration of a father or mother is affecting or has affected you? How often and in what ways do you communicate with your father or mother who has migrated? For example by phone, Skype, Facebook?
Healthy Boundaries (parental discipline of youth)
Q3. When you need advice, who do you tend to go to, your father or mother? Why do you think this is? Does this change when the father migrates? Q4. When you are disciplined by either of your parents, who do you pay more attention to? Why do you think this is? Does this change when the father migrates?

### 2.5 Data analysis

We applied Bowlby’s (1980) attachment theory as the analytic lens through which to understand disruptions in attachment in children of migrant families and the consequences of disrupted attachment in the context of parental migration (24). According to Bowlby’s attachment theory, individuals have an internal working model (IWM) of themselves formed by early parent-child emotional relationships that influence subsequent development. This internal working model is used to cope with stress, regulate emotions, and develop close relationships throughout one’s life (24). The three primary attachment categories are: secure, avoidant, and anxious. Children with a secure attachment to their parents are more likely to exhibit psychological well-being with adaptive coping strategies, emotional regulation skills, and high self-efficacy. Insecure attachments occur when the child experiences avoidant or anxious attachment styles with their caregiver and, in turn, develops a negative internal working model of themselves and others. Children who have experienced a history of rejection or caregiver inconsistency by their adult caregivers are at high risk of developing insecure attachment relationships characterized by avoidant and anxious relationships with themselves and others. Individuals with insecure attachments will exhibit high levels of anxiety and avoidance that result in the use of maladaptive coping strategies, a lack of emotional regulation skills, fear of abandonment, exaggerated emotional and behavioral responses, and difficulty forming close relationships. During coding, we applied attachment theory principles to contextualize youth sentiments and behaviors in the context of parental migration.

We applied principles of thematic analysis to guide data analysis (25). The lead authors (KM and MLZ) identified initial codes and developed a final coding scheme for the 23 interviews. The study’s senior investigator is a bilingual (Spanish and English) and bicultural Latina with expertise in Mexican migration and is an experienced translator. A systematic examination of the transcripts, including both initial reading and in-depth re-reading, was conducted by the second author (KM) with close oversight by the study’s senior investigator who also conducted field interviews (MLZ). The initial coding list was generated by KM with ongoing weekly discussion with the study’s senior investigator (MLZ), who reviewed coded documents. With ongoing refinement of the coding list, a final list of codes was compiled. Following this process, the second author (KM) coded the transcripts and the first author (MLZ) reviewed the coded transcripts, with continued discussion of additional coding refinements as warranted. The units of textual records that were coded were then organized into thematic axes of analysis, following a consensus among all researchers regarding the highlighted quotations selected. We applied attachment theory and utilized the Social-Ecological Model (SEM) to identify and understand community perspectives surrounding individual, interpersonal, community, and societal factors that may place youth whose parents have migrated at risk for poor mental health, substance use, and other risky behaviors.

## 3 Results

A total of 12 youths were interviewed, eight females and four males. Two of the youth were siblings who lived with both parents and were interviewed independently at different times. At the time of the interview, youth were an average 18 years of age (SD 1.3). Additional demographic information is found in **Table 1**. Of note is that at the time of the youth interview, a majority of fathers lived in the state of Yucatán in locations within approximately 3 hours distance by bus or car, with only one father currently living in the U.S. and one father in Mexico City. Parental migration details such as the number of migrations, duration away from the family were not collected as part of this study and are discussed in the limitations section.

We found that parental migration, both domestically and internationally, impacted youth mental health and substance use in a myriad of ways. Overall, youth, parents, and teachers perceived that parental migration created insecure attachment relationships for youth with both their migrant and non-migrant parent, or extended family (e.g., parent, aunt, or grandparent), and this insecure attachment influenced youth risk behavior, including substance use. Alcohol and marijuana appeared to be the most commonly used substances among youth, with some reports of early age of alcohol consumption (12 or 13 years).

We identified six major migration-related themes in this study: (1) Parent attachment and challenges faced by parents in supervising children and children’s unmet emotional needs; (2) Youth risk behavior as attention seeking when a parent migrates; (3) Adverse youth mental health outcomes as a result of parental migration; (4) Youth drinking to cope “*Para Olvidarse de los Problemas*” (to forget one’s problems); (5) Unintended consequences of remittances as facilitators of access to alcohol and drugs; and (6) Protective factors and youth resilience. Following, we expand upon each theme, providing context and perspectives from youth, parents, and teachers.

### 3.1 Parent-child attachment, child supervision, and unmet emotional needs of children

A primary theme raised by students, parents, and teachers alike was the strained relationship/bond between parents and children and the limited parental attention, guidance, affection and supervision paid to the youth as a result of parental migration. Mothers whose spouses had migrated expressed difficulty raising their children alone and felt that children’s behaviors changed with the father’s absence. Reflecting on her partner’s numerous migrations and returns from California over the course of 20 years, one mother described how this dynamic affected her, her children, and child supervision:

“Well the truth is that it is a difficult change because the children become accustomed to one authority, they became used to just me….In reality, from a young age, my children wanted to have their father close. But when they turned 9 years old, they started to go with their little friends. And I had their younger siblings [to care for] and with the chores, I could not go out looking for them. From there they started to have a certain greater liberty than what they should have at their age. But because I was by myself. I could not. I think that for this reason, these are the factors that my children, one could say because I have two boys, that they now smoke marijuana.” *mother #1*

Youth considered the impact of parental absence on them, including new limitations on their ability to communicate with parents and how parental absence affected parent-child attachment and the family overall:

“They leave and well that affects your family because everything shifts … it’s an absence that you feel from your mom or your dad, whoever is not there and well you sometimes want to tell them something and like they say, it would always be better for it to be your mom or dad, but if they’re not there you lose … you can even stop caring for them.” *female youth* #5

In response to interview questions surrounding youth communication with a parent who had migrated, they shared that communication was primarily through cell phone calls, texts, WhastApp and Facebook. Frequency of communication was sometimes variable, ranging from daily to weekly contact. Youth participants largely welcomed these calls and said it made them feel happy.

Parents and teachers also reported noticing a change in children’s behavior as a result of their father migrating and the child losing his “cariño” (affection), which created unmet emotional needs:

“Initially there was a change in her, because she was very attached with [sic to] her dad. She had behavior problems then. She would fight a lot and then isolated herself from her peers. This was the problem. She does have her own character, sometimes isolates herself from others because she rebels a lot … My family is not the kind that shows a lot of love, myself included. And I think that my daughter is lacking this. I stop myself and put a barrier between myself and them. I’m not the type to show them affection. I took her to a psychologist, and she told me that she was lacking affection, that is the problem, and I think that this is what pushes her to do things that she shouldn’t do.” mother #8

### 3.2 Youth risk behavior as attention seeking when a parent migrates

Youth reflected on how their risk behavior changed towards risk behaviors in order to seek parental attention. One student discussed what motivated her to engage in regular drinking behavior:

“[I thought] that maybe by acting like that I wanted to see or bring my family together again, so that my dad would say, “Oh no, my daughter is not doing well.” I don’t know; I was trying to get my dad’s attention sometimes, and sometimes because of the depression, I wanted to settle my body and my mind in my addictions.” *female student #12*

One of the teacher focus group participants echoed the same perception:

“The youth do not have the affective part (attention, self-esteem, interest). So they try to get their attention in the sense that … I’m going to do drugs and bad things as a call for attention, so that somebody will come and say “what’s going on?”

Another student describes how the youth’s loneliness drives them to drink, how she lacks guidance and advice from her parents, and how her own sadness and happiness are dependent upon visitations with her parents when they return some weekends to see her:

“Their parents leave and they feel alone because they’re gone, they don’t know what to do so they start to drink and take drugs … A lot of youth are missing their dads, in my case, we are missing them because there’s nobody to give us advice, my sister is not the same as my mom or dad, your mom can advise you, tell you what’s good and bad and how to resolve problems. Sometimes you feel alone because you don’t have them. Or when they do come you’re happy and when they leave again you feel sadness.*” female student #9*

### 3.3 Adverse youth mental health outcomes as a result of parental migration

A theme that was present in almost every interview was how children struggled with depression, “*tristeza” (*sadness*)*, loneliness, and anxiety as a result of their parents migrating:

“If you are very attached with your dad it affects you a lot. If your dad had never left town for work and was always with you, when he leaves it affects you, You don’t want to eat, you’re willing to do anything in order to forget.” *female student #7*

Others reflected on how their *tristeza* affected them longer term, including experimentation with substances:

“I was 11 when he left [to the U.S.]. I cried for him, I wanted him to stay. I felt like a huge part of me was leaving, I felt like I had no dad … I thought how is it possible that my dad is alive but not with me in the most important events of my life, the changing stages of my life … it’s not fair, I’m fortunate that God gave me a dad that has been alive until now and he’s not with us, it is as if he were dead. Yeah, it makes me really angry and sad … Sometimes we fall into a depression and we go to other people and lots of times to the vices.” *female student* #12

“In the same way sadness enters, since one’s parents are not present, and well they do not have anyone they can go to. And well at the same time they start to feel sad and well several others say: ‘drink so that you don’t feel sad’” *male student #1*

### 3.4 Youth drinking to cope *para olvidarse de los problemas* (to forget one’s problems)

Alcohol consumption was commonly reported, with some youth beginning to drink by age 12 or 13 years. Some participants described children as young as 11 exhibiting problematic substance use behaviors such as huffing paint thinner and gradually using other drugs like cocaine and crack. Alcohol consumption appeared to be part of maladaptive coping, as indicated by one student who discussed peer influence on drinking to cope with their sadness:

“The sadness enters and since their parents are not there, well they don’t have anyone to turn to and … at the same time they feel sad and lots of their friends will tell them “drink so that you don’t feel sad, so that this can all pass” well they end up starting to drink … I’ve heard this, “drink so that you don’t feel sad” or “drink to relieve your stress” and well, that’s how the youth fall into it.” *male student #1*

When asked why they believe that youth drink more when their parents are gone, one mother said:

“Because they are alone and some say that they don’t have anyone. It is like therapy for

them. But not because it affects them more.” *mother #7*

Youth substance use also manifests as a result of feelings of parental betrayal or feeling left behind. We observed expressions of this insecure attachment and substance use in many interviews. For example, a father with a migration history that we spoke with conveyed to us the sheer impact that the migration of a parent can have on their child:

“They start with cigarettes and end with marijuana, that’s how they fall into addiction. Primarily because they think they were betrayed when their parents left them and because they feel alone. Loneliness is bad company … [they turn to the vices] because they feel abandoned and cheated. Because sometimes they were told: I’ll be gone a month and come back for you, and after years pass, 5 years and nothing. They are left all alone. They feel betrayed and abandoned.” *father #2*

### 3.5 Unintended consequences of remittance as facilitators of access to alcohol and drugs

Remittances and material gifts sent home by the migrant parent were mentioned during interviews. These financial resources supported family needs such as furniture and allowed youth to purchase items such as clothing. Youth also received computer tablets or phones, distinguishing them from their peers who did not have access to resources from remittances or material goods. Some youth reported that this greater relative affluence also facilitated youth access to alcohol and drugs.

“The town festival is in August and there you see who does not have their dad here but has the money to spend on drinking and everything.” This same student later emphasized: “In the cantinas (the youth) pawn their phone for beer and then once you’ve got the money you give it to them and they give it back to you. Whatever it is, rings, watches, they accept anything there … as long as you drink there. A youth cannot get something if they don’t have money or if they don’t have a phone or tablet or something … it’s something that their dad also gives them, he sends it and they pawn it then say “oh, I lost it” and their mom just says oh my son lost it to the dad and says “send another because our son lost it” and he’ll send another.” *female student #5*

### 3.6 Protective factors and youth resilience

Our analysis revealed two elements that appear to positively influence children’s mental health. One is the presence of strong parent-child attachments, while the other is the emotional support provided by teachers. Strong parent-child attachments were evident through various parenting strategies aimed at steering youth away from risky behaviors. These strategies include maintaining open communication, regularly checking in with teachers, offering additional school support, and serving as positive role models. One mother we interviewed explained:

“We have always talked about everything with our children, the good and the bad. The consequences of taking drugs, drinking, prostitution. You have to talk about everything with them, you know? I have talked to my children a lot, and I explain things to them with videos on the internet and TV. There is a lot of communication between them and us and I think that has helped them not to veer off track and to stay out of trouble. (…)

With respect to their education, I go to their school often. Any little problem—I am there and I also ask for their grades. I support and help them a lot with school. I think that because of the trust that they have in me, they will come and tell me anything. (…)

Also, here in the home we do not consume anything, and since they do not see it at home, they do not try to imitate it.” *mother #3*

Teachers’ emotional support played a role in motivating students to seek help to deal with alcohol use. A youth that we interviewed who was in recovery from alcoholism explained:

“I go to AA [Alcoholics Anonymous] self-help group every weekend, and when you feel bad, they will do daily groups in the evening, and there you can talk to someone and get the weight off, then return home. (…) Before, I would not go to anyone, but now I have a friend who is my teacher here; she has been helping me a lot psychologically.” *female student #12*

Collectively, these perspectives underscore youth and parent resilience in the face of stressors and health impacts related to parental migration, youth’s ability to seek resources for treatment and self-care, and insight into what their community can do to support youth and engage them in alternative, alcohol-free activities and environments.

## 4 Discussion

This study revealed important findings about the critical role that parental migration plays in youth access to and risk for the use of alcohol and marijuana. We found that, as a result of parent migration, youth who remain at home experienced emotional hardships (e.g., depression and sadness) that led to maladaptive coping strategies such as engaging in alcohol and/or drug use. Disruption within the family system due to migration, along with the unpredictable nature of visitations and communication from migrant parents, resulted in insecure attachments among their children that also placed them at risk for substance use. Our findings are aligned with two recent studies looking at the impact of migration on communities left behind in Latin America. Carranza and colleagues (2022) (12) and Ciborowski and colleagues (2022) (13) also found profound negative impacts on youth health related to emotional well-being, lack of parental attention, and substance use.

Our findings also underscore the potential duality of remittances as a benefit to families but with the potential unintended consequences of facilitating youth access to alcohol through material goods that can be pawned for access or financial resources to purchase substances. This youth’s access to alcohol is exacerbated when combined with a lack of supervision from the remaining parent or other caregiver. To date, a limited understanding of factors related to parental migration that drive substance use risk among youth remaining in their community of origin is an area of needed research to which the current study contributes. We consider study implications and concurrence with the existing literature by primary theme.

### 4.1 Parent attachment and challenges faced by parents in supervising children and children’s unmet emotional needs

Youth, parents, and teachers themselves identified attachment issues as being highly correlated with drinking and drug use behaviors among the Tunkaseño youth. Our findings showed that many youths were having trouble developing a positive internal working model of themselves due to insecure attachments to their parents. For example, according to attachment theory, when a father says he will return but does not, that child may lose the ability to trust others. The youth in our interviews described the migration of their parent(s) as an extreme hardship that was detrimental to their emotional well-being. The attachment difficulties that resulted from parental migration were shown in various ways to impact the healthy development of youth in Tunkás.

These findings are in alignment with our framework on child attachment. According to Bowlby (24), when a caregiver is inconsistent in their actions or presence, children are at risk of developing an insecure attachment that is associated with fears of abandonment and feelings of anxiety. Youth whose parents migrated identified irregular communication and visitations with their parents as saddening and anxiety-provoking. This insecure attachment can lead to exaggerated emotional reactions and feelings of abandonment, as evidenced in our interviews. Youth, parents, and teachers identified children’s unmet needs for attention, guidance, affection, and/or supervision as a reason for their increased rates of alcohol and drug consumption. Several respondents articulated that children feel so lonely and desperate for their parent’s attention that they have turned to drug and alcohol consumption as a cry for help. Following the work of Song and Glick (2022), future research should employ a life-course perspective to understand the complexities surrounding parental migration and opportunities for intervention with youth and families more comprehensively (26).

### 4.2 Youth risk behavior as attention seeking and adverse youth mental health outcomes as a result of parental migration

Youth depression and loneliness, frequently mentioned across participant interviews, are well documented in the literature among children who remain after parental migration, although there are inconsistent findings on the nature and degree of impact of migration on mental health across studies (10). In their meta-analysis comparing children and adolescents with and without parents who had migrated, Fellmeth and colleagues (2018) found an elevated risk of depression, conduct disorder, and substance use among those with migrating parents, although no differences were found relating to other indicators of wellbeing (e.g., nutrition, unintentional injury, and abuse): Importantly, the significance of substance use risk varied across studies (9). Our study was not designed to capture the complexities of drivers of migration (e.g., reasons for migration, number of migrations, where parents migrated, duration of each trip and nature and frequency of contact with children and family). Although further study is needed, we would hypothesize that for some migrants there may be multiple reasons for migration, including economic and family discord. Indeed, because circumstances surrounding parent migration are multifaceted and complex, future studies should be longitudinal and include metrics of child and family risk and well-being, including measurement of and statistically controlling for child pre-existing risk for substance use *prior* to parent migration. Studies should also consistently include standardized measures of alcohol and substance use risk to more fully understand the nature and prevalence of substance use and the mechanisms through which parental migration influences substance use and other risk behaviors, as well as youth resilience (e.g., educational attainment). Data on adolescent and adult mental health in Tunkás was not located, however, Ramírez-Toscano, et al. (2023) (27) study on alcohol consumption patterns in Mexican adolescents finds that the prevalence of current drinking and heavy binge drinking in past 12 months was about 21% and 14%, respectively, indicating the need for greater prevention interventions among adolescents. Importantly, school enrollment figured prominently as a study finding: adolescents who were not attending school had a higher alcohol use risk profile (27). This finding underscores the importance of school engagement and the potential protective factor that school can offer youth as we saw in our study.

### 4.3 Youth drinking to cope “Para Olvidarse de los Problemas” (to forget one’s problems)

Maladaptive coping is a common behavior for people who did not learn to create a positive internal working model of the world through their parental attachments and therefore have not learned to properly regulate emotions and cope with stress (24). We observed maladaptive coping strategies throughout our interviews in the form of excessive drinking by youth “to forget about their problems.” A theme voiced by many participants in our study was the use of alcohol and other drugs to cope with problems, to de-stress, and to avoid *tristeza* (sadness). Youth are frequently exposed to peer and parent messages that drinking helps you “forget about your problems,” but in actuality, youth reflected that drinking made their problems worse or made them feel sadder. This finding may indicate a clear attempt at managing youth’s emotional reactions to the loss of a parent with maladaptive coping strategies.

Our research found high levels of self-reported depression among the youth who were dealing with the emotional impact of their parent’s migration. Some youth even described the traumatic experience of losing their parent(s) to international migration as emotionally equivalent to their parent’s death. Early loss or separation and parental rejection (ie: feeling betrayed and abandoned) are all risk factors caused by a parent’s migration that may lead to higher rates of risky behavior in youth (14).

### 4.4 Unintended consequences of remittance as facilitators of access to alcohol and drugs

The novel findings relating remittances to high-risk behavior should be further evaluated in a future study to better understand the conditions under which remittances can have a positive and negative impact on child health. The economic benefit of remittances to children and families who remain when a parent migrates, including higher household resources compared to children with non-migrant family circumstances, has been documented (5, 10). Given the enormous economic role of remittances in Mexico and globally, longitudinal research with youth remaining at home may improve our understanding of the conditions and context of benefits and risks to children and families receiving remittances. In their systematic literature review of child well-being among children left behind, Antia and colleagues (2020) found that remittances from parents who migrate can promote materialism among youth and that remittances also tend to be used fully for family needs and did not necessarily contribute to improved child socioeconomic conditions (10). In our study, however, remittances, in the form of cash and goods, played an important part in youth access to substances, which is a novel finding. Goods included electronics such as phones, tablets, and computers. We also found that remittance money given directly to youth often served to increase their disposable income and ability to purchase alcohol and other drugs for themselves and their peers.

Because underage drinking laws were reportedly not uniformly enforced by the town’s stores or cantinas, youth with cash and goods were able to access alcohol relatively easily. This informal system implicates adult providers of alcohol to underage minors, which exacerbates already easy access to alcohol. Local policy enforcement to curtail bars selling alcohol to minors and other efforts to limit access to alcohol among youth in Tunkás are worthy of community attention. The environmental availability of alcohol in small towns such as Tunkás is important to note. Our prior work with the Tunkás community indicates a ubiquitous availability of liquor in Tunkás and communities where members migrate, which influences consumption patterns among migrants (28, 29). Furthermore, an earlier study conducted by a member of our Investigator team (Dr. Pedro Lewin) indicated that the female head of household whose partner had migrated to the U.S. or elsewhere in Mexico expressed facing stressors such as weakening of their marital relationship, marital difficulties and emotional instability among younger children after their spouses migrated, further indicating the potential negative health impact on the family (30).

### 4.5 Protective factors and youth resilience

The primary protective factors identified in the study were the presence of both parental figures, strong parent-child attachment, good communication between parents and youth, parental supervision and monitoring, parental support and responsiveness, and strong teacher emotional support for students. Some students described their teachers as the most important adult figures in their lives, and one in particular shared that she goes to her teacher for psychological support. The school provides an important protected space for students to seek support from teachers and lends itself as the driver of future interventions that can improve student resilience as they face significant life challenges, such as parental migration. Therefore, it is crucial that future interventions and efforts to support youth resilience be focused on and disseminated in the school setting.

Research on traditional Mexican cultural values, such as “*familismo*” (familism) and collectivism also provide evidence for protecting youth and families from greater harm when facing challenges or stressful events, such as the migration of a parent. In Parmar et al. (2023), a qualitative study with Latina immigrant mothers with children living in a rural community in California, the authors describe protective factors to reduce community-level adverse childhood experiences (ACEs) and intergenerational trauma (31). Mothers described the central role of the family in supporting child health, including good communication, respect, and affection. Spirituality and caring for community members were also among key findings as factors of resilience among Latino families. Additional protective activities employed by the mothers included parental engagement with clinicians to link their children with mental health support, parent-child communication towards planning for education and a promising future and long-term positive outcomes, and parents serving as role models for self-care and resilience (31). Drawing from the literature on resilience and post-traumatic growth, we find additional insights on potential interventions for children of migrants in the work of Powell and colleagues (2021) (32) who conducted a systematic review of coping and post-traumatic stress in children and adolescents after a disaster. We learn that higher child resilience, the belief in one’s ability to cope, was weakly correlated with lower rates of PTSD in children with trauma and that routines in families and schools were also inversely associated with PTSD symptoms (32). Similarly, Wen and colleagues (2021) found that the role of families and schools is central to the health of children: their analyses indicated that social capital in family and school settings accounted for differences in self-efficacy between children with parents who migrated in comparison with children of parents who had not migrated. Lower self‐efficacy was associated with poorer relationships with parents and schoolteachers (33)

## 5 Limitations

Important limitations and strengths of this study are worthy of consideration. As an exploratory qualitative study, our findings cannot be generalized to the experiences of youth in other communities that have experienced high parental migration; this includes youth in other parts of Mexico and Latin America. If parental migration occurred early on in the youths’ lives, participant recall bias may influence their recollection of the experience. Our interviews indicate, however, that some face lingering trauma as a result of parental migration, which underscores the impact on current youth mental health and substance use behavior and the need to implement interventions that bolster protective behaviors and health. Our study did not include detailed information about parental migration patterns or dates and length of time away. We asked youth participants about the current location of their parent who migrated. Because migration in many families in Tunkás frequently includes domestic and international movement at different times by the same member of the family, we are unable to comment on whether the current location/residence of the parent is the only migration event for that parent nor can we say with certainty which migration instance or instances the youth may be recalling. For the two siblings who were interviewed and living with both parents, probing for their experience with migration may have yielded additional information. Indeed, more detailed information about migration history and current circumstances and relationships with parents would provide a more nuanced understanding of the potential variation in impact of migration on youth and should be included in future studies.

That the respondents were mainly mothers is worthy of note and may introduce a bias where the paternal perspective was not well represented in our study. We note, however, that majority interviews with mothers may reflect that the fathers were working or living elsewhere at the time of the survey, as indicated by youth responses to where their fathers were living at the time of the interview. Since mothers are the primary caregivers when the male partner migrates, mothers’ perspectives are valuable to understand different factors relating to family response to migration such as changes in maternal responsibilities, ability to supervise children, and administration of family resources. Inclusion of paternal perspectives is warranted, and future studies could outreach to the male partners and invite them for virtual interviews in order to gain a more comprehensive perspective on the impact of migration on the family, as well as document migration timing and patterns. Locating fathers in domestic and international settings would further expand our understanding of the impact of migration on youth and families. Longitudinal studies on parental migration with more detailed information on the nature and frequency of contact with youth could also provide important information about the proximal and longer-term impact of parental migration on youth health.

The study does not capture the participants’ ability to consider the complexity of parental migration, both positive and negative, and how it impacted them. Although our interview guide questions (**Table 2**) were written to allow for a breadth of response (i.e., positive or negative experiences with migration), we did not specifically probe for positive experiences. This is a potential source of bias that we are unable to provide a more comprehensive understanding of youth and parent participant experiences and elicitation specifically of both types of experiences is important for subsequent migration research.

The study site is a rural Mexican town that may not represent the experiences of youth in urban settings. As one of the few studies to focus specifically on factors that may be driving substance use risk and the experiences of youth remaining in rural communities, however, this study contributes to our understanding of the nature of challenges among rural youth and may inform subsequent intervention research to support youth resilience and wellbeing. This study provides information that can inform subsequent needed research in the context of parental migration in Latin America, as there remains a significant research gap from the Americas on youth risk for alcohol and substance use and prevalence of use in the context of parental migration. Of the 14 studies that included youth risk and substance use metrics in Fellmeth and colleagues’ systematic review and meta-analysis (2018), most were studies from China, and none were based on data from Latin America (9). Similarly, among the 30 studies included for a systematic review looking at economic migration and its impact on the mental health and well-being of children left behind, Antia and colleagues (2020) found only three studies from Latin America, all from Mexico (10).

Including youth, parent, and teacher voices also enriches our understanding of perspectives on similar issues, underscoring the potential for problem-solving to reduce youth risk through collaboration across the community. Longitudinal studies that include youth with migrating parent(s) and youth with no parental migration will aid in an improved understanding of youth risk behavior. Future research should also continue to engage community members in the research process and identify possible solutions. This will elevate the meaningfulness of research findings to communities to improve child health and resilience in the context of ongoing parental migration.

The study relied on a convenience sample of youth and parents who were available and willing to share their experiences with us and for this reason is limited in representation of community parents and youth in Tunkás. However, our engagement with high school teachers who had worked almost a decade with youth and parents in Tunkás, provided an important perspective that triangulated with findings from youth and parent interviews. Related, we did not follow a systematic assessment of theoretical saturation of data, however, we feel that the study provides important perspectives that reveal youth and parent experiences that can inform future studies. Also, the study was not designed to capture information from participants whose parents did not migrate and this limitation should be addressed in future research in order to understand similarities and differences in substance use among youth in the community. Similarly, we are unable to comment on other stressors that may be influencing youth alcohol consumption and poor mental health in addition to experiences of parental migration, this is also an important area of research for future study.

And finally, although we do not have systematically gathered information on the drinking/drug culture and norms of Tunkás community members, researchers’ extensive experiences and observations in the community at the time of interviews and community feedback sessions lead us to believe that alcohol is easily accessible in Tunkás and that this availability may play a role in drinking culture. For example, beer is widely available in small family-owned shops and the presence of cantinas (bars) and heavy use among some members of the community was observed by investigators, especially during town fiestas.

## 6 Conclusions, public policy recommendations, and community follow up

Our study underscores the profound emotional impact that parental migration has on parent-child attachments and gives insight into how insecure child-parent attachment relationships in the context of parental migration may place youth at risk for engaging in alcohol and drug consumption in communities with high parental migration. These factors should be considered in the design of future intervention research to support youth facing parental migration and improve youth resilience to negative physical and mental health outcomes in other communities where parental migration is common. Our interviews also revealed that community members are interested in interventions targeting youth access to alcohol and substance use in their community. Although 18 years of age is the legal drinking age in Mexico, public health policy and efforts to reduce drinking (binge drinking in particular) and access to alcohol among underage youth should continue to be supported across communities in Mexico. Recent research by Colchero and colleagues on binge drinking in Mexico (2022) (34), including underage consumption, identified the problematic increase in binge drinking and environmental risks including higher density of places that sell alcohol and lower prices of alcohol as contributors to binge drinking (34). Their research supports calls for improved public policies to reduce binge drinking through higher taxation of alcohol and reduced density of bars, stores, and other venues that sell alcohol (34).

Our study findings have important implications for youth health, and taken with existing parental migration research, they may inform a better evidence base for Mexican migration policy. Our study identified parental support, guidance, and advice as necessary to support healthy coping behaviors for youth. New policies are needed to comprehensively address youth mental health and resources that parents and youth can access, as needed, to help the family cope more effectively with parental migration. Because the CBTA school was found to be an important point of convergence for students, teachers, and parents alike, interventions could benefit from using the school setting as a starting point to support youth.

One policy approach to promoting family well-being in the context of parental migration could be providing psychoeducation classes for parents about the impact of parent-child attachment on youth’s emotional and physical well-being. This type of programming may include evidence-based approaches to manage youth and parent emotions and prepare families for the departure of a parent. Drawing from evidence-based practices towards family resilience in the face of parental absence, such as in the case of preparing military families for the deployment of a military parent (35), research with military family experiences may provide ideas for families preparing for parental migration. Other avenues may include training teachers and community leaders to serve as peer support providers to support parents and youth in high-migration communities like Tunkás. As noted in this study, youth with lived experiences and insight into the impacts on them and their families, when a parent migrates, should play an active role in working with their communities to develop interventions to improve youth health and well-being. As of this writing, teachers and representatives from the CBTA High School with whom the research team remains in contact have confirmed that many of the problems faced by their students with migrant parents still exist. They have expressed interest in continued work with the research team to discuss findings, identify potential interventions, and together develop a plan that could be implemented by the school to better support youth with a parent who has migrated or will migrate.

## Data availability statement

The datasets presented in this article are not readily available because author preference is to not make qualitative interview transcripts publicly available due to potential for identification of community members from the small indigenous community where the study was conducted. Requests to access the datasets should be directed to mlzuniga@sdsu.edu.

## Ethics statement

The studies involving humans were approved by Human Research Protection Program, San Diego State University. The studies were conducted in accordance with the local legislation and institutional requirements. Written informed consent for participation in this study was provided by all participants and if participant was under 18 years of age, consent was provided by the participant's legal guardian.

## Author contributions

MZ: Writing – review & editing, Writing – original draft, Visualization, Validation, Supervision, Project administration, Methodology, Investigation, Funding acquisition, Formal analysis, Data curation, Conceptualization. KM: Writing – review & editing, Writing – original draft, Methodology, Funding acquisition, Formal analysis, Conceptualization. PL-F: Writing – review & editing, Validation, Supervision, Project administration, Investigation, Formal analysis, Data curation, Conceptualization. IM: Writing – review & editing, Validation, Investigation, Formal analysis, Data curation. LT: Writing – review & editing. LU: Writing – review & editing, Conceptualization.
